# Annexin gene family in *Spirometra mansoni* (Cestoda: Diphyllobothriidae) and its phylogenetic pattern among Platyhelminthes of medical interest[Fn FN1]

**DOI:** 10.1051/parasite/2024034

**Published:** 2024-06-21

**Authors:** Xiao Yi Su, Fei Gao, Si Yao Wang, Jing Li, Zhong Quan Wang, Xi Zhang

**Affiliations:** Department of Parasitology, School of Basic Medical Sciences, Zhengzhou University Zhengzhou 450051 PR China

**Keywords:** Tapeworm, Plerocercoid, Annexin family, Molecular characterization, Evolutionary pattern

## Abstract

The plerocercoid larvae of *Spirometra mansoni* are etiological agents of human and animal sparganosis. Annexins are proteins with important roles in parasites. However, our knowledge of annexins in *S. mansoni* is still inadequate. In this study, 18 new members of the Annexin (ANX) family were characterized in *S. mansoni*. The clustering analysis demonstrated that all the SmANXs were divided into two main classes, consistent with the patterns of conserved motif organization. The 18 SmANXs were detected at all developmental stages (plerocercoid, adult, and egg) and displayed ubiquitous but highly variable expression patterns in all tissues/organs studied. The representative member rSmANX18 was successfully cloned and expressed. The protein was immunolocalized in the tegument and parenchyma of the plerocercoid and in the tegument, parenchyma, uterus and egg shell of adult worms. The recombinant protein can bind phospholipids with high affinity in a Ca^2+^-dependent manner, shows high anticoagulant activity and combines with FITC to recognize apoptotic cells. Annexin gene polymorphism and conservative core motif permutation were found in both cestodes and trematodes. SmANXs also revealed high genetic diversity among Platyhelminthes of medical interest. Our findings lay a foundation for further studies on the biological functions of ANXs in *S. mansoni* as well as other taxa in which ANXs occur*.*

## Abstract (fr)

Les larves plérocercoïdes de *Spirometra mansoni* sont des agents étiologiques de la sparganose humaine et animale. Les annexines sont des protéines jouant un rôle important chez les parasites. Cependant, nos connaissances sur les annexines chez *S. mansoni* sont encore insuffisantes. Dans cette étude, 18 nouveaux membres de la famille des annexines (ANX) ont été caractérisés chez *S. mansoni*. L’analyse de regroupement a démontré que tous les SmANX étaient divisées en deux classes principales, ce qui correspond aux modèles d’organisation des motifs conservés. Les 18 SmANX ont été détectées à tous les stades de développement (plérocercoïde, adulte et œuf) et présentaient des modèles d’expression omniprésents mais très variables dans tous les tissus/organes étudiés. Le membre représentatif rSmANX18 a été cloné et exprimé avec succès. La protéine a été immunolocalisée dans le tégument et le parenchyme du plérocercoïde ainsi que dans le tégument, le parenchyme, l’utérus et la coquille d’œuf des vers adultes. La protéine recombinante peut se lier aux phospholipides avec une affinité élevée de manière dépendante du Ca^2+^, présente une activité anticoagulante élevée et se combine avec le FITC pour reconnaître les cellules apoptotiques. Un polymorphisme du gène de l’annexine et une permutation conservatrice du motif central ont été trouvés chez les cestodes et les trématodes. Les SmANX ont également révélé une grande diversité génétique parmi les Plathelminthes d’intérêt médical. Nos résultats jettent les bases pour des études plus approfondies sur les fonctions biologiques des ANX chez *S. mansoni* ainsi que dans d’autres taxons dans lesquels les ANX sont présents.

## Introduction

Sparganosis is a neglected foodborne and waterborne zoonotic disease caused by the plerocercoid larvae (sparganum) of various diphyllobothroid tapeworms belonging to the genus *Spirometra*, which causes losses to socioeconomic development and poses a threat to public health [8, 23, 52]. Human beings are mainly infected via the consumption of undercooked meat derived from frogs or snakes infected with plerocercoids, through drinking untreated water containing *Spirometra* larvae, or by placing poultices of frog or snake flesh on open wounds [12, 20]. Sparganosis typically manifests as migrating larvae, and the symptoms depend on their localization in the body. After infecting humans, the ingested sparganum can invade the subcutaneous tissues, spinal cord, eyes, breasts and brain, resulting in local tissue damage, paralysis, blindness, and even death [10, 31]. Human sparganosis has been reported in Asian, African, American and European countries, with more than 2000 cases to date [1, 3, 13, 23, 35]. China has the largest number of sparganosis cases in the world, with more than 1300 cases reported in 27 out of 34 provinces, autonomous regions, or municipal districts [32]. Although the diagnosis of sparganosis involves laboratory tests, imageological and molecular techniques have been introduced, and strategies such as surgery and drugs are treatment options; however, due to very weak molecular biological knowledge of *Spirometra mansoni*, precision medicine treatment for sparganosis still has a long way to go [9]. Therefore, addressing this knowledge gap is an urgent matter.

Annexin (ANX) is a type of calcium-dependent phospholipid binding protein that forms an evolutionarily conserved multigene family with members expressed in eukaryotic cells across all kingdoms [16, 43, 50]. Structurally, annexins are characterized by a highly alpha-helical and tightly packed protein core domain, which consists of four so-called annexin repeats, and each repeat contains a ‘type 2 or 3’ motif considered to represent a Ca^2+^-regulated membrane binding module [7, 48]. Because of their binding to Ca^2+^, annexins can participate in a series of membrane biological activities, including vesicular transport, signal transduction, formation of calcium channels, regulation of inflammatory response, cell differentiation and apoptosis, and interaction with cytoskeletal proteins [6, 36]. According to their amino acid conservation and phylogenetic inferences, the ANX superfamily can be classified into five categories: annexin A–E [37]. Family A mainly occurs in vertebrate cells, including 12 identified members (annexins A1–A11, A13) and an unassigned member (A12); annexins outside vertebrates are classified into families B (invertebrates, including parasitic helminths), C (fungi and some groups of unicellular eukaryotes), D (plants) and E (protists). Nevertheless, there are at least 40 unclassified members to date. Owing to their important role in host-parasite interactions and their immunogenic properties, ANXs are attractive as targets of new anthelminthic drugs or vaccines [19]. Since the first ANX was reported in 1977 [37], annexins have been identified in a large variety of helminth species, such as *Angiostrongylus cantonensis*, *Taenia crassicep*, *Taenia multiceps*, *Echinococcus granulosus* and *Schistosoma mansoni* [18, 29, 42, 45, 46]. Furthermore, comparative genomic and transcriptomic analyses showed that the annexin family was highly expressed in *Spirometra* tapeworms [33], indicating the crucial roles of ANXs in *S. mansoni*. Knowledge of the structure, molecular characteristics and evolutionary pattern of the annexin family will be helpful for understanding host–parasite interactions, as well as developing new intervention strategies for sparganosis. However, no ANXs have ever been studied in *Spirometra* species thus far, and we know nearly nothing about the molecular features of ANXs in this tapeworm of medical concern.

In this study, we intended to screen ANX members in *S. mansoni* (SmANXs) based on all available -omic databases, investigate their molecular characteristics, and explore the phylogenetic patterns of SmANX members in medical Platyhelminthes. More specifically, the following aims were addressed: (1) to investigate protein family members, gene expression patterns and molecular characterizations of ANX in *S. mansoni*, and (2) to investigate the gene organization and evolutionary fate of ANXs across parasitic Platyhelminthes.

## Materials and methods

### Ethics approval and consent to participate

This study was approved by the Life Science Ethics Committee of Zhengzhou University (No. 2021-0916).

### Samples and experimental animals

The *Spirometra* plerocercoid larvae were originally isolated from wild infected frogs in Zhengzhou city, Henan province, China. The collected plerocercoids were tentatively identified as *S. mansoni* using molecular typing method described in Kuchta [23]. The scolex of plerocercoids were orally administered to female specific pathogen-free (SPF) mice (3 plerocercoids per mouse) for serial passage in our laboratory. Positive serum against plerocercoid was obtained from the conserved mice. Twenty female BALB/c mice were used to immunize by recombinant annexin (rANX) four times to obtain the anti-rSmANX sera. All anti-rSmANX sera were stored at −80 °C until use. Additionally, an adult worm representing *S. mansoni* was obtained from an infected domestic cat using a previously described method [9]. The immature proglottid (IP), mature proglottid (MP) and gravid proglottid (GP) from adult worms were collected according to their position and morphology. All samples were snap-frozen in liquid nitrogen after washing 3 times using physiological saline, and then stored at –80 °C for subsequent use.

### SmANX family member identification

Candidate sequences that contain the annexin protein domain were searched using the NCBI Conserved Domains database (www.ncbi.nlm.nih.gov/Structure/cdd/wrpsb.cgi). All candidate SmANXs were obtained from the WormBase ParaSite database (https://parasite.wormbase.org/) and recently published transcriptomic data [33]. These extracted sequences were identified as members of the ANX family through querying for genes annotated with the Pfam domain accession pfam00191. All screened candidates from multi-omic data were analyzed using the HMMER tool (https://www.ebi.ac.uk/Tools/hmmer/) to confirm the presence of the conserved annexin domain [40]. In addition, for candidate SmANXs retrieved from the transcriptomic data, the nucleotide sequences were first translated to amino acid sequences using the NCBI’s ORF finder tool (https://www.ncbi.nlm.nih.gov/orffinder/) and BLASTX for homology searches. Finally, these retrieved sequences were corroborated by cloning and sequencing of *S. mansoni* DNAs. The basic physical and chemical properties of all identified SmANXs were predicted using the ExPASy (https://www.expasy.org/). The subcellular localization was predicted by TargetP (www.cbs.dtu.dk/services/TargetP/). The conserved protein motif analysis was performed using the mixture model by the expectation maximization (MEME) method (https://meme-suite.org/meme/tools/meme), and the gene features were performed using GSDS. Motif scan and the NCBI-CDD server (https://www.ncbi.nlm.nih.gov/Structure/cdd/wrpsb.cgi) were used for conserved functional protein domain prediction. Multiple sequence alignments of SmANXs were carried out with DNAMAN software. The phylogenetic tree was inferred using the maximum likelihood (ML) method based on the GRT + G model. The ML analysis was performed in MEGA v7 [25] with 1000 bootstrap replications. Secondary structure was generated by the PSIPRED server (http://bioinf.cs.ucl.ac.uk/psipred/). The 3D structure was determined using homology modeling available at the Swiss Model server (https://swissmodel.expasy.org/), and the quality of the model was examined using Ramachandran plot analysis and visualized by the Swiss-PdbViewer v.4.1 [21].

### Expression patterns of SmANXs

The expression levels of SmANXs in three life cycle stages of *S. mansoni*: plerocercoid stage, adult (including immature proglottid, mature proglottid and gravid proglottid) and egg were monitored using a quantitative real-time PCR method. The gene-specific primers are listed in Supplementary Table S1. Total RNA was isolated using a reverse transcription kit (Novoprotein, Shanghai, China). qRT-PCR was conducted on a 7500 Fast Real-time PCR system (Applied Biosystem, Monza, Italy). The reaction mixture contained 10 μL of 2 × SYBR^®^ Green Pro Taq HS Premix (ROX plus) (Accurate Biology, Hunan, China), 10 μM each of sense and antisense primers, 100 ng of first-strand cDNA. Initial thermal-cycling at 95 °C for 30 s followed by 40 cycles of 95 °C for 3 s and 60 °C for 30 s. The GAPDH gene served as the internal control [33]. Relative gene expression levels were analyzed according to the comparative 2^−∆∆Ct^ method [27].

### Cloning and expression of rSmANX

The SmANX18 was selected for cloning and expression to explore the molecular characteristics. PCR amplification was carried out with the cDNA of plerocercoid. The gene was amplified by PCR with specific primers carrying BamHI and HindIII restriction enzyme sites (underlined) (forward, 5ʹ-GCGGATCCTTTGTGCTTAGTCTC-3ʹ, and reverse, 5ʹ-GCAAGCTTAGAACGATTATGATGAGG-3ʹ). The cycling protocol was as follows: 35 cycles of 94 °C for 30 s, 53 °C for 30 s and 72 °C for 30 s. The PCR products cloned into pMD19-T vector and pMAL-c2X expression vector in turn after gel recovery. The recombinant plasmid was then transformed into *Escherichia coli* BL21 (New England Biolabs, Ipswich, MA, USA). Expression of rSmANX 18 was induced by adding 0.5 mM IPTG at 33 °C for 4 h. rSmANX18 purification was completed by affinity chromatography using an amylose resin column (ShengGong, Shanghai, China) and identified by sodium dodecyl-sulfate polyacrylamide gel electrophoresis (SDS-PAGE). Images of gels were recorded using Image Scanner (GE Healthcare, Fairfield, CT, USA).

### Indirect ELISA development

Antibody titrations of immunized mice were detected by indirect ELISA. The rSmANX18 protein (0.5 μg/mL) was coated onto each well of 96-well plates overnight at 4 °C and then blocked with 200 μL of phosphate-buffered saline 0.1% Tween 20 (PBST) containing 5% skimmed milk. Immune serum (100 μL) with serial dilutions was added to each well and incubated at 37 °C for 2 h. HRP-labelled goat anti-mouse IgG (EarthOX, Millbrae, CA, USA) was added at a 1:5000 dilution and incubated for 1 h at 37 °C. Finally, 100 μL OPD chromogen substrate containing H_2_O_2_ was added to each well; the plates were incubated for 15 min, and the reaction was stopped by the addition of 50 μL 2 M H_2_SO_4_. The optical density (OD) of all wells was measured at 490 nm using a computer-controlled microplate reader (BioTek Synergy LX, Santa Clara, CA, USA).

### Indirect immunofluorescence assay

Indirect immunofluorescence assay (IFA) was used to locate the positions of the target gene. Eggs, tissue sections of plerocercoids and different proglottids of adult worms were first retrieved after microwaving for 20 min with a 0.01 M citric acid buffer (pH 6.0), blocking with 5% normal goat serum in PBS, then incubated with a 1:10 dilution of anti-rSmANX18 serum, serum of mice infected with plerocercoids, and normal mouse serum in PBS at 37 °C for 1 h, respectively. The sections were incubated with a 1:50 dilution of FITC-labeled anti-mouse IgG (Santa Cruz Biotechnology, Dallas, TX, USA), and the nuclei were stained with propidium iodide (PI) at 37 °C for 15 min. Finally, the sections were examined under a fluorescent microscope (Olympus, Tokyo, Japan) after washing 6 times with PBS.

### Phospholipid-binding bioactivity assay

Liposomes were prepared as described previously [46]. The phospholipid-binding assay was based on previous reports with some modifications [30]. To determine the calcium ion-dependent phospholipid membrane binding characteristics of rSmANX18, three experimental groups (A–C) and two control groups (D–E) were used. Twenty microliters of liposomes, 30 μL of rSmANX, and 30 μL of 1 mM CaCl_2_ (except for Group C) were added to each experimental group, and Group D was established with 20 μL of liposomes, 30 μL of MBP protein, and 30 μL of 1 mM CaCl_2_. Group C was the same as Group D except for CaCl_2_. Next, 50 mM Tris–HCl was added for every group as a supplement to a total volume of 100 μL. All groups were incubated in ice water and centrifuged to separate the supernatant from the precipitate. The precipitate in Group B was washed with Tris–HCl. Thirty microliters of 1 mM ethylenediaminetetraacetic acid (EDTA) and 70 μL of Tris–HCl were added to the precipitate of Group B. The supernatant and precipitate were separated by centrifugation. All the supernatant and precipitate samples were analyzed using 10% SDS-PAGE and estimated densitometrically using ImageJ Analysis Software. Each assay was independently assessed three times.

### Anticoagulation activity test and thrombolytic experiment of annexin *in vitro*

Annexin has been confirmed to play important roles in the coagulation process [38]. The anticoagulant activity of rSmANX18 was analyzed with two coagulation tests: general screening of the coagulation time (CT) test and activated partial thromboplastin time (aPTT) test. Specifically, the slide method was used to measure the blood coagulation time [2]. Blood collected from 12 h fasted mice was mixed with different concentrations (0–1.5 mg/mL) of rSmANX18 solution, and the mixed liquor was placed onto a slide. Then, the mixed liquor was stirred continuously in the slide using a needle and observed regularly until the presence of fibrin. The CT test was controlled at 25 °C with humidity of 60%. Clotting time was manually recorded and analyzed using one-way ANOVA. Meanwhile, the anticoagulant activity of rSmANX18 was assayed using an aPTT method as reported previously [15]. In brief, first, 0.1 mL of normal mixed plasma and 0.1 mL of aPTT reagent (Shenzhen Zike Biotechnology, Shanghai, China) were preincubated at 37 °C for 3 min. Next, normal mixed plasma (100 μL), different concentrations of rSmANX18 (100 μL) (0–600 μg) and ellagic acid reagent (100 μL) were mixed and incubated at 37 °C for 5 min. Finally, 100 μL of 25 mM CaCl_2_ was added to allow the initiation of clot formation, and the time required for formation of a stable clot was recorded simultaneously. Equivalent volumes of normal saline and MBP protein were used as controls. Moreover, a thrombolytic test was conducted *in vitro* to explore the thrombus formation ability of annexin. Cotton threads were placed into the blood from infected mice to make thrombus strips. Then, incubated thrombus strips were placed into solutions containing rSmANX18, normal saline and MBP tag proteins at 37 °C for 24 h. The degree of thrombus strip dissolution and solution color changes were observed by photography.

### Apoptosis detection of mammalian tumor cells

Fluorescein isothiocyanate (FITC)-labelled annexin was used as a fluorescent probe to recognize apoptotic cells through binding of FITC−ANX to phosphatidylserine (PS) exposed at the external surface of the cell membrane [26]. Specifically, FITC labelling was achieved by dialyzing purified rSmANX18 against 25 mM sodium bicarbonate buffer (pH 9.0). Dialyzed rSmANX18 (50 μM) was mixed with 50 μM FITC (Yeason, China) and incubated in sodium bicarbonate buffer at 4 °C for 24 h. The labelled mixture was dialyzed against Tris buffer and subsequently placed in PBS buffer to separate labelled protein from free FITC [54]. The final concentration of FITC-labelled SmANX18 was adjusted to 10 μg/mL and stored in the dark at 4 °C. For the assessment of FITC-SmANX18 binding to apoptotic cells, two apoptotic cell models were established. The RAW264.7 cell line was used to test 10% ethanol-induced apoptosis. Serum-free culture-induced apoptosis with the Jurkat T lymphocytic cell line (ATCC TIB 152) was also investigated. All cell suspensions were cultured at a density of 1 × 10^6^ cells per milliliter in RPMI 1640 (Servicebio, Wuhan, China) supplemented with 10% fetal calf serum (EVERY GREEN, Zhejiang, China), 100 μg/mL streptomycin, and 100 IU/mL penicillin. Double staining for FITC–annexin 18/PI or FITC–annexin A5 (annexin V)/PI binding was performed as follows: after two washes in cold PBS, 1 × 10^5^ cells were resuspended in binding buffer. Then, 5–10 μL FITC–annexin 18 or 5 μL FITC–annexin V was added to 100 μL of the cell suspensions (1 × 10^5^ cells). To distinguish cells that had lost membrane integrity, 5 μL propidium iodide (PI; Yeason) was added before flow cytometric analysis. The mixture was incubated for 15 min in the dark at room temperature and then subjected to quantitative analysis by flow cytometry (BD FACS Calibur, San Jose, CA, USA) within one hour [24, 34].

### ANX sequence retrieval from other Platyhelminthes

The ANX sequences of 27 other helminths of medical interest (Supplementary Table S2) were extracted from the WormBase ParaSite and NCBI database using the key word “Annexin” based on the following criteria: synteny, the presence of a single exon in most sequences (or the conserved position of the intron where present), and phylogenetic relationships between orthologs. For each species, we blasted each ANX protein sequence against the entire genome, applying an e-value threshold of 1e−1, and using opening and extending gap penalties of 14 and 2, respectively. The expected maximization mixed model (MEME) was used for ANXs motif analysis [4]. Protein sequences were aligned with MAFFT v7 [22].

### Phylogenetic analysis

Phylogenetic analyses were performed using two methods: Bayesian inference (BI) and maximum likelihood (ML), respectively. Protein sequences were aligned with MAFFT v7 using the FFT-NS-I method, and any columns containing more than 95% gaps were deleted using Gap Strip/Squeeze v2.1. The ML tree was implemented in PhyMLv3.0 [17], and branch support was calculated with aLRT-SH method. The best substitution model was defined with the Smart Model Selection (SMS) tool [28] incorporated in PhyML. The Bayesian inference (BI) tree was constructed with a run length of 50 million generations and sampling every 5000 generations by using BEAST v1.8.4 [14]. The software TRACER v1.6 was used to check the convergence of Monte Carlo Markov Chains (MCMC) and to ensure adequate effective sample sizes (ESS > 200) after the first 20% of generations were deleted as burn-in. The maximum clade credibility tree was estimated with TreeAnnotator, which is part of the BEAST v1.8.4 package, and the tree was visualized using Figtree v1.4.3.

## Results

### Manual annotation of SmANX genes

In *S. mansoni*, 18 sequences containing ANX domains were identified by screening (Table 1). All obtained sequences were deposited in GenBank under accession numbers SmANX1 to SmANX18. Among SmANXs, 17 were identified as intracellular ANXs, and 1 belonged to the secretory annexin family (SmANX6). Among the 17 intracellular ANXs, 11 were identified as cANXs (cytoplasmic ANXs), and 6 were mANXs (mitochondrial ANXs). Fourteen A types (SmANX1-SmANX7, SmANX9-SmANX11 and SmANX13-SmANX16), 2 B types (SmANX8 and SmANX12) and 2 E types (SmANX17 and SmANX18) were classified. The length of SmANXs ranged from 390 bp to 2187 bp. The predicted protein length ranged from 125 aa to 419 aa. In the case of domain length, the length ranged from 41 aa to 73 aa. The molecular weights varied from 14,028.07 Da to 44,682.07 Da, while the theoretical isoelectric points ranged from 4.79 to 9.36.

**Table 1:** Annotation features for *Spirometra mansoni* annexins.

Gene name	CDS (bp)	Protein (aa)	ANX domain coordinates	Domain length (aa)	Mw (Da)	PI	Subcellular location
SmANX1	855	284	1–48, 56–128, 216–281	44, 71, 64	31694.09	4.87	M
SmANX2	1245	370	24–88, 96–139, 220–292, 302–367	64, 65, 72, 43	41611.17	5.77	other
SmANX3	1012	323	26–91, 99–171, 187–245, 255–320	65, 72, 58, 65	36084.26	4.91	other
SmANX4	540	170	1–42	41	18881.30	6.58	other
SmANX5	390	125	11–76, 83–125	65, 42	14028.07	5.21	other
SmANX6	534	177	34–99, 109–174	65, 65	19732.80	9.20	S
SmANX7	767	209	59–125, 135–200	66, 65	23896.32	9.10	other
SmANX8	1398	419	121–186, 194–258, 276–342, 352–417	65, 64, 66, 65	44484.55	8.35	other
SmANX9	675	223	73–139, 149–214	66, 65	25306.95	9.36	M
SmANX10	1086	355	62–127, 134–206, 238–304	65, 72, 66	39819.38	8.88	other
SmANX11	1305	367	55–120, 128–200, 232–298	65, 72, 66	41064.97	6.36	M
SmANX12	2187	356	93–158, 166–230, 248–314	65, 64, 66	37937.20	8.36	other
SmANX13	922	274	41–114, 130–196, 207–271	73, 66, 64	30425.51	5.87	other
SmANX14	1014	336	43–108, 115–187, 219–285	65, 72, 66	37920.33	9.11	M
SmANX15	1310	376	55–120, 128–200, 232–298, 308–373	65, 72, 66, 65	41892.76	5.40	M
SmANX16	1117	319	14–78, 87–159, 175–241, 252–316	64, 72, 66, 64	35275.28	6.15	other
SmANX17	1290	396	73–138, 146–218, 249–316, 326–390	65, 72, 67, 64	44682.07	6.12	other
SmANX18	1065	354	31–96, 104–176, 208–274, 284–348	65, 72, 66, 64	40168.03	6.28	M

### Features of SmANXs

**Figure 1:**
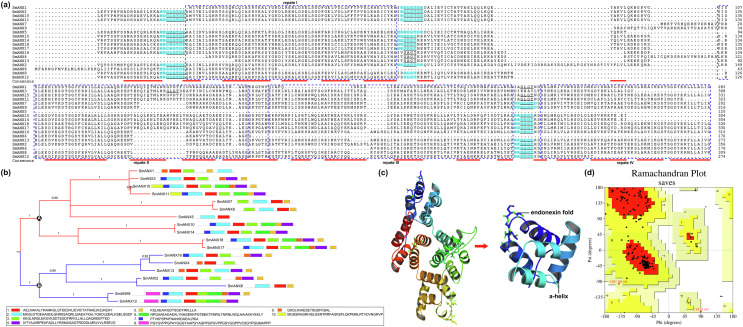
Annexin protein family members identified in *Spirometra mansoni*. (a) Multiple alignment of protein sequences of SmANXs; predicted α-helices are indicated in red. The four-repeat domains of the annexin sequence are indicated with blue dotted boxes. (b) Phylogenetic tree and conserved motifs of SmANXs. (c) Visualization of the SmANX protein model. Each color represents one “annexin repeat”, and each annexin repeat consists of 5 α-helices containing calcium ion binding sites between the fourth and fifth α-helices, called the endonexin fold. (d) Model quality evaluation of the 3D structure of SmANX. Red represents the most favored regions, yellow indicates additional allowed regions, pink indicates the generously allowed regions, and light color indicates disallowed regions.

Multiple alignment analysis showed that the core domain contained four internal repeat units (repeats I-IV), and each repeat harbored type II and III Ca^2+^-binding sites (Fig. 1a). According to phylogenetic analysis of full-length protein sequences, SmANXs could be categorized into two main clades: Clade A and Clade B (Fig. 1b). Clade A consisted of all mANXs and 5 cANXs. The remaining cANXs and a sANX (secretory annexin) were within Clade B. The MEME program determined 10 specific putative motifs that contained 21–50 residues (Fig. 1b and Supplementary Table S3). The motif scan analysis revealed that SmANXs contained 1~4 typical annexin motifs of calcium ion-dependent phospholipid binding sites (type II or type III calcium-binding site) known as the “endonexin fold”. The 3D structural homology model showed the formation of typical N-terminal and C-terminal domains in SmANX3 (Fig. 1c). SmANX can be divided into four internal repeat units, called “annexin repeats”, and each repeat unit contains five α helices, termed A–E, which fold into a domain in the form of a right-handed superhelix. Helices A, B, D and E were oriented pairwise in an antiparallel fashion, while helix C was positioned as a bridge orthogonally to the others. The Ramachandran plot analysis showed that 91.5% of the residues (260 aa) were located in the favored region, 7.7% of the residues (22 aa) were located in the allowed region and only 0.7% of the residues (2 aa) were located in the outlier region, suggesting high quality of the protein model (Fig. 1d).

### Relative expression of SmANXs

**Figure 2:**
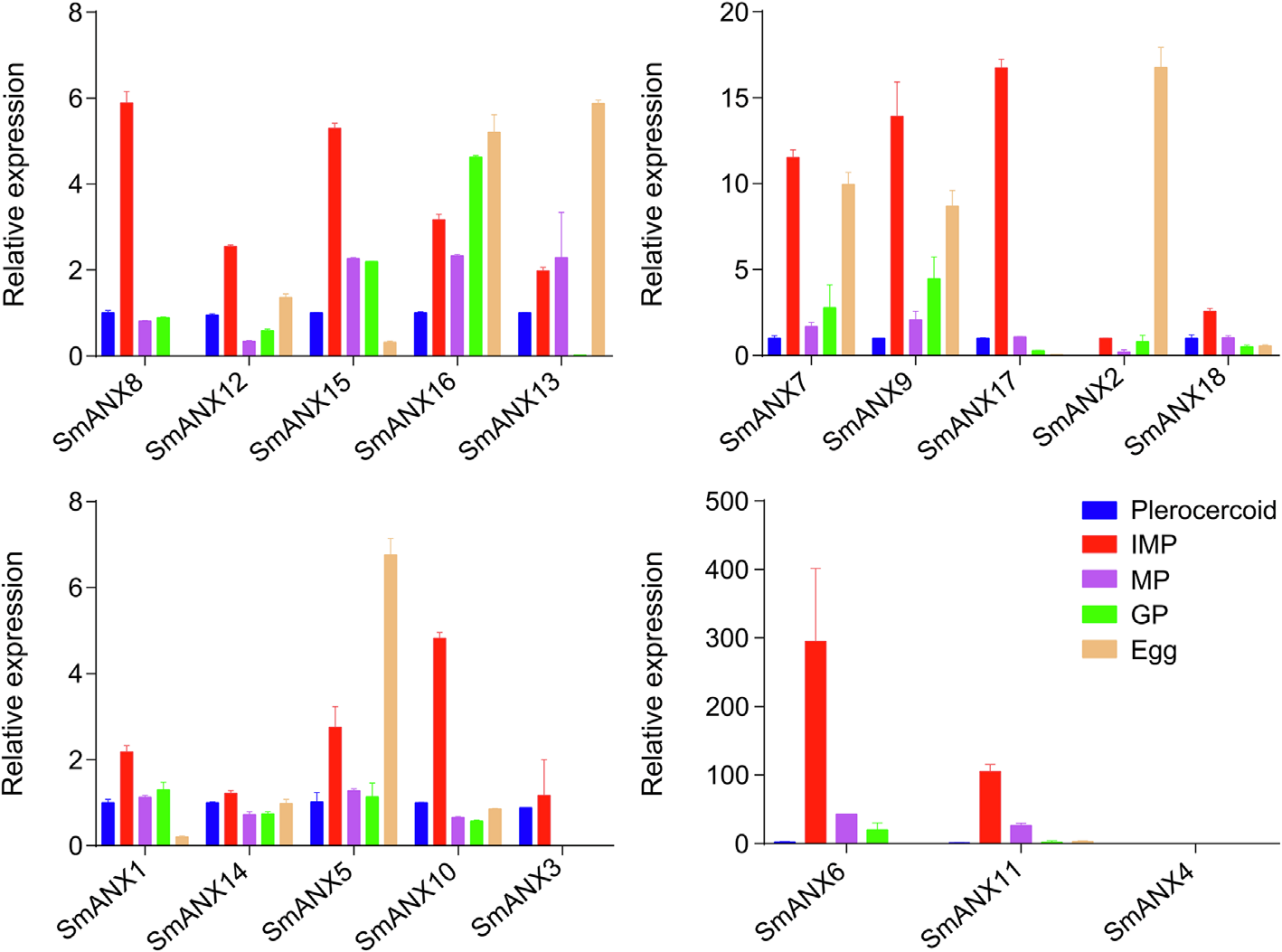
ANX gene expression of *S. mansoni* in different stages by qRT-PCR. The expression level was normalized to that of GAPDH and measured with the 2^−ΔΔCt^ method. The results were averaged from three independent replicates during all stages. Error bars represent SD (*n* = 3). IMP, immature proglottid; MP, mature proglottid; GP, gravid proglottid. Blue represents plerocercoid; red represents IMP; purple represents MP; green represents GP; orange represents egg.

To profile the expression patterns of identified SmANXs, we sampled eggs, plerocercoids, and different proglottids of adults for analysis by qRT–PCR (Fig. 2). A total of 13 SmANXs were expressed in the egg stage, 16 in the larval stage, and 17 in the adult stage. In the egg stage, 6 genes were highly expressed, while only 2 were highly expressed in the plerocercoid stage. In the adult stage, 9 genes were highly expressed. Among these, 8 genes were highly expressed in the immature proglottid, 2 genes were highly expressed in the mature proglottid, and only 3 genes were highly expressed in the gravid proglottid.

### Molecular characterization of SmANX18

**Figure 3:**
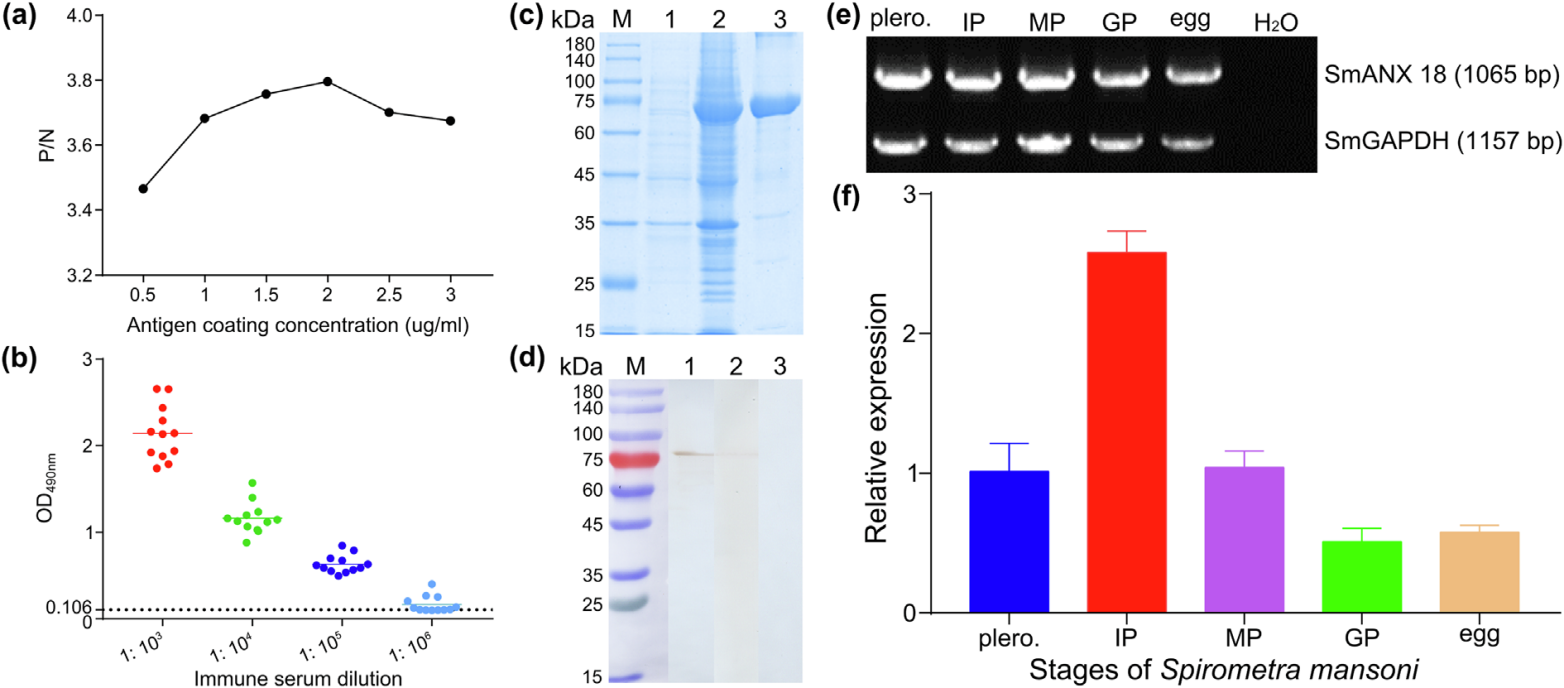
Molecular characterization of cloned SmANX 18. (a) Determination of the optimal antigen coating concentration. (b) Determination of anti-rSmANX18 immune serum titer by indirect ELISA. Red, green, blue, and light blue represent serum dilutions of 1:10^3^, 1:10^4^, 1:10^5^, and 1:10^6^, respectively. (c) SDS-PAGE analysis of 10 μg purified ANX from *S. mansoni* on a 10% gel. M: protein prestaining marker; Lane 1: uninduced bacterial cultures; Lane 2: the lysate of the induced recombinant bacteria harboring PMAL-c2X-rSmANX 18 after ultrasonication; Lane 3: rSmANX 18 purified by amylose resin column. (d) rSmANX 18 antigenicity analysis. M: protein prestaining marker; Lane 1: rSmANX 18 + anti-rSmANX 18 serum; Lane 2: rSmANX 18 + infected mouse serum; Lane 3: rSmANX 18 + preimmune serum. (e) The transcription pattern of the ANX gene in different developmental stages of *Spirometra mansoni* by conventional RT-PCR. The cDNA from various developmental stages of *S. mansoni,* including eggs, plerocercoid and immature proglottids, mature proglottids, and gravid proglottids of adult worms. A housekeeping gene (Sm-GAPDH) was used as a positive control. H_2_O was used as a negative control. (f) rSmANX 18 expression of *S. mansoni* in different stages by qRT-PCR. Blue represents plerocercoid; red represents IMP; purple represents MP; green represents GP; orange represents egg. IMP, immature proglottid; MP, mature proglottid; GP, gravid proglottid. The expression level was normalized to GAPDH and measured with the 2^−ΔΔCt^ method. The results were averaged from three independent replicates during all stages. Error bars represent SD (*n* = 3).

**Figure 4:**
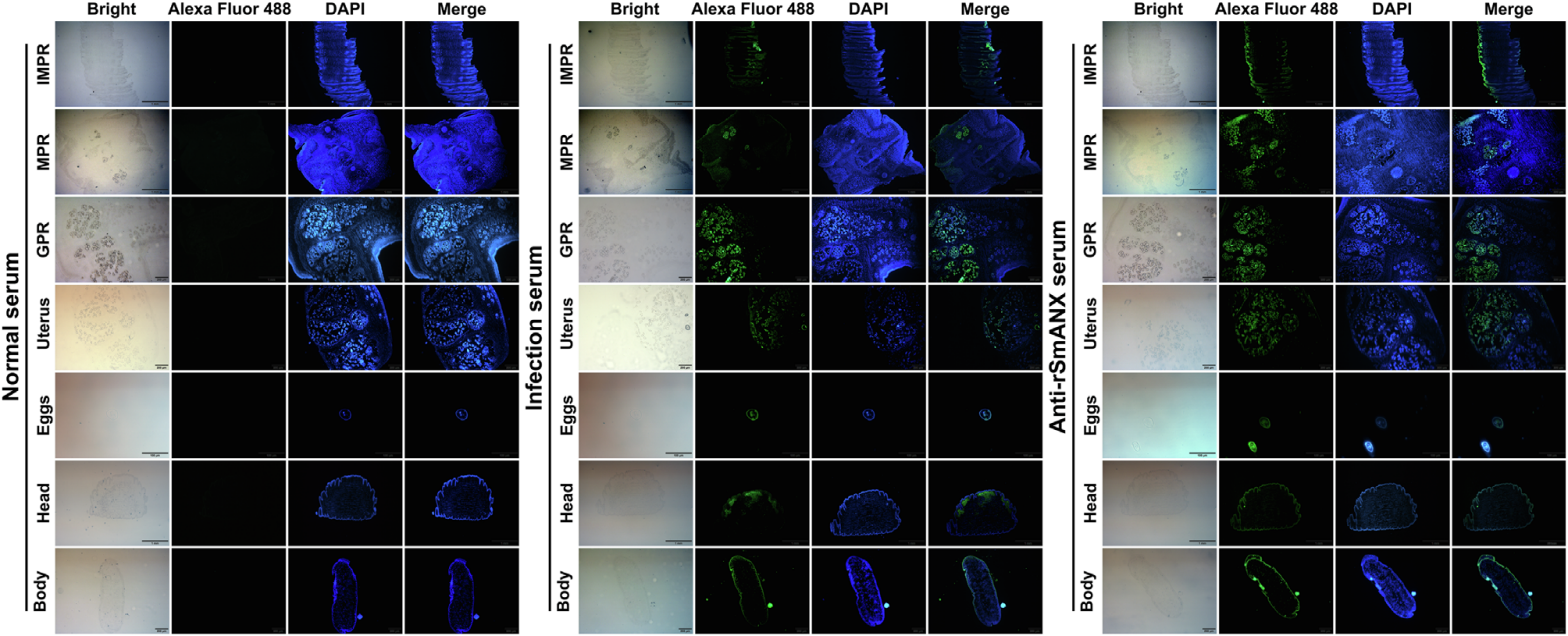
Immunofluorescence localization of ANX in different developmental tissues of *Spirometra mansoni*. The plerocercoid tissue contained the head and body, and the adult worm contained IMPR, MPR, GPR, uterus, and eggs. IMPR indicates immature proglottid, MPR indicates mature proglottid, GPR indicates gravid proglottid. Green fluorescence (Alexa Fluor 488) indicates the location of the ANX protein. Scale of IMPR, MPR, and head of plerocercoid: 1 mm; GPR, uterus, and body of plerocercoid: 200 μm; eggs: 100 μm.

**Figure S1:**
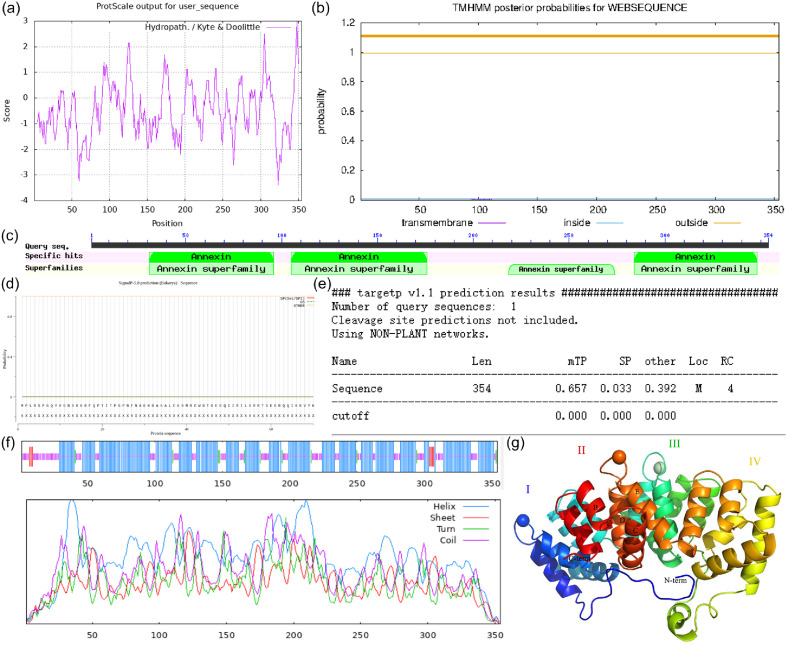
Prediction of basic physical and chemical properties of SmANX 18. (a) hydrophobicity prediction; (b) transmembrane prediction; (c) functional domain prediction; (d) signal peptide prediction; (e) subcellular localization prediction; (f) secondary structure prediction; (g) 3D structure prediction.

Molecular biological analysis showed that SmANX18 is a cellular protein with a predicted Mw of 40.168 ku and a pI of 6.28 containing 4 calcium ion-dependent phospholipid binding sites (Supplementary Fig. S1). SmANX18 was cloned into the prokaryotic expression vector pMAL-c2X. The recombinant plasmid pMAL-c2X-SmANX18 expressed a soluble fusion protein. Meanwhile, an indirect ELISA based on the rSmANX18 protein was established. A protein concentration of 2 μg/mL and a mouse serum dilution of 1:100 were the optimal conditions (Fig. 3a). The cut-off value of 0.106 was used as a standard for the subsequent tests (Fig. 3b). The molecular mass of rSmANX18 was approximately 80 kDa, which was close to the expected value (the pMAL-c2X tag proteins weigh approximately 40 kDa) (Fig. 3c). The concentration of rSmANX18 was 2 mg/mL. Western blotting analysis showed that rSmANX18 was recognized by the serum of mice infected with plerocercoids as well as the anti-rSmANX18 serum (Fig. 3d). The mRNA transcription of the SmANX18 gene was observed at the egg, plerocercoid and different proglottid stages in adults (Fig. 3e). The qPCR analysis showed that the transcriptional level of the immature proglottid was the highest, followed by the mature proglottid and plerocercoid (Fig. 3f). The immunolocalization test showed that specific fluorescence staining was observed in egg shells, plerocercoids and adults. In the plerocercoid, fluorescence staining was detected subcutaneously. In the adult stage, fluorescence staining was observed in the tegument of each proglottid, as well as in the parenchyma. At a higher magnification, the tissues and follicles around the uterus in the mature or gravid proglottid were stained green, which indicated that annexin was highly expressed in the uterus (Fig. 4).

### Phospholipid-binding bioactivity analysis

**Figure 5:**
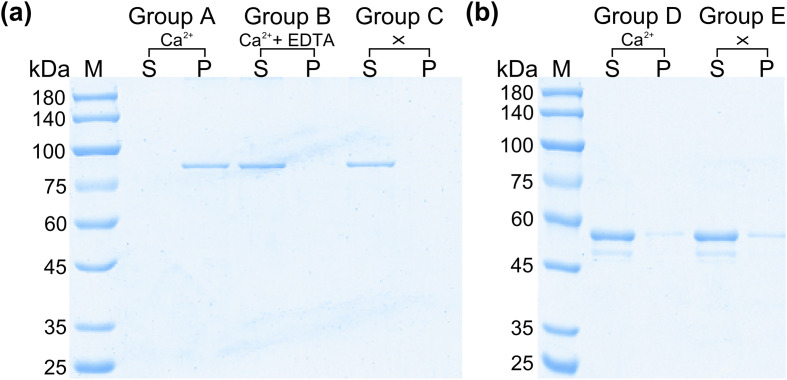
Phospholipid-binding properties of rSmANX18. (a) Phospholipid-binding properties of rSmANX18. Lane *M* = molecular mass marker in kDa; Group A = rSmANX18 incubated with liposomes in buffer containing 1 mM Ca^2+^; Group B = rSmANX18 incubated with liposomes in buffer containing 1 mM Ca^2+^ and 1 mM EDTA was then added; Group C was the control group (no Ca^2+^ or EDTA). (b) The control experiment of MBP protein. Lane *M* = molecular mass marker in kDa; Group D = MBP protein incubated with liposomes in buffer containing 1 mM Ca^2+^; Group E = MBP protein incubated with liposomes (no Ca^2+^ or EDTA). Ca^2+^ + EDTA (mM) = the concentrations of added Ca^2+^ and EDTA, respectively; *P* = pellet; *S* = supernatant.

rSmANX18 was allowed to react with lipidosomes in the presence or absence of Ca^2+^ to estimate their calcium-dependent phospholipid-binding properties. In the presence of Ca^2+^, rSmANX18 can bind phospholipids and was observed in the precipitate (Group A). When Ca^2+^ was removed using EDTA, rSmANX18 was released from the precipitate into the supernatant and no longer bound phospholipids (Group B). In the absence of Ca^2+^, rSmANX18 was observed in the supernatant (Group C) (Fig. 5a). The control groups showed that the MBP protein was mainly detected in the supernatant in the presence (Group D) or absence (Group E) of calcium ions (Fig. 5b). The results verified that rSmANX18 can bind to phospholipid membranes with the participation of calcium ions.

### Anticoagulant activity analysis of rSmANX

**Figure 6:**
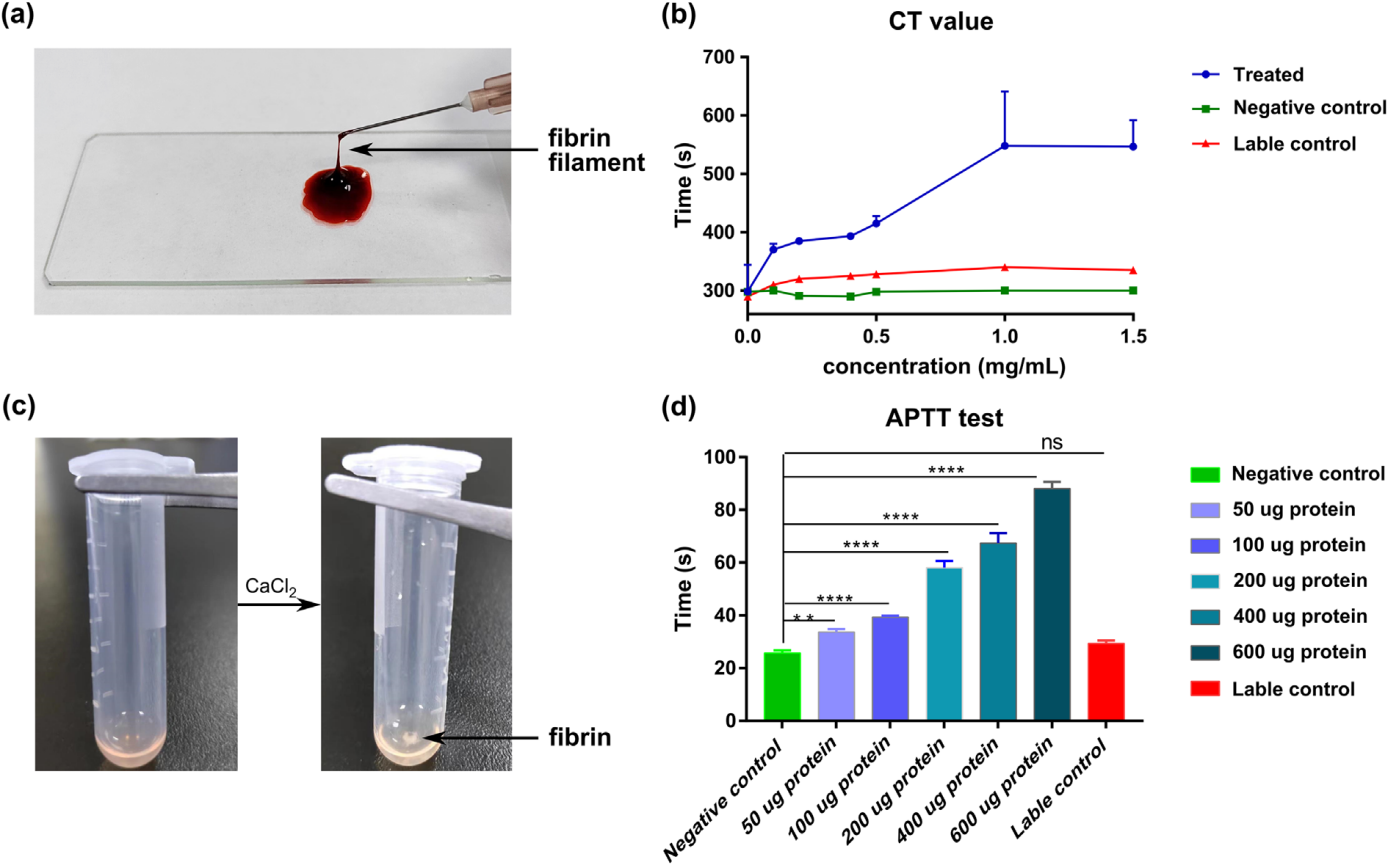
Anticoagulant activity of SmANX 18. (a) The formation of fibrin filaments in blood coagulation. (b) Analysis of the anticoagulant activity of SmANX 18 by the coagulation time test (CT). Normal saline and MBP protein were used as controls. Each point is the mean of two replicates ± standard deviation. (c) Changes in the plasma state during blood coagulation. Fibrin was formed after adding calcium ions. (d) The anticoagulant activity assay of SmANX 18 by aPTT. Normal saline and MBP protein were used as controls. The experiments were performed three times. ****p < 0.001, **p < 0.05, ns represents no significant difference.

The CT assay showed significant differences between the negative control (normal saline), label control (MBP protein) and experimental groups (containing different concentrations of rSmANX18) (Table 2). Consistent with the CT assay, a significant difference (*p* < 0.001) was observed between the experimental group and the controls (MBP protein-labelled group and normal saline group). However, no significant differences were detected between the normal saline group and MBP group in either CT or APTT. The slide method test (Fig. 6a) revealed that the coagulation time of the rSmANX18 group (0–600 μg) was longer than that of the normal saline group as well as the MBP group at each concentration, indicating that rSmANX18 possessed the ability to reduce blood clotting. As shown in Figure 6b, in comparison with the average 300 s clotting time of the negative control, samples mixed with different concentrations of rSmANX18 had more prolonged coagulation times. The coagulation time was prolonged with increasing rSmANX18 concentration, especially when the concentration exceeded 0.5 mg/mL. The coagulation time peaked value at a concentration of 1 mg/mL. The APTT assay (Fig. 6c) also supported the significant anticoagulation activity of rSmANX18. As shown in Figure 6d, the coagulation time peaked value (88 s) at an rSmANX18 dosage of 600 μg. In comparison with MBP protein and normal saline, rSmANX18 prolonged the clotting times in a concentration-dependent manner. Both CT and APTT methods validated that rSmANX18 can prolong the clotting time *in vitro*, indicating significant anticoagulant activity of SmANX.

**Table 2:** Determination of coagulation time by the slide method.

Slide method	Solute concentration	Clotting time (seconds)	
		Baseline	Treatment* (Mean ± SD)
Negative control (normal saline)**	0.9%	300	296.7 ± 4.7
Label control (MBP)*	5 mg/mL	290	326.7 ± 9.7
Experimental groups* (rSmANX 18)	0.1 mg/mL	300	370 ± 7.1
	0.2 mg/mL	290	385 ± 4.1
	0.4 mg/mL	290	390 ± 4.1
	0.5 mg/mL	295	415 ± 10.8
	1 mg/mL	300	548.3 ± 37.9
	1.5 mg/mL	305	546.7 ± 36.8

** Highly significant difference.

* Significant difference.

SD: standard deviation.

### Apoptosis assay

**Figure 7:**
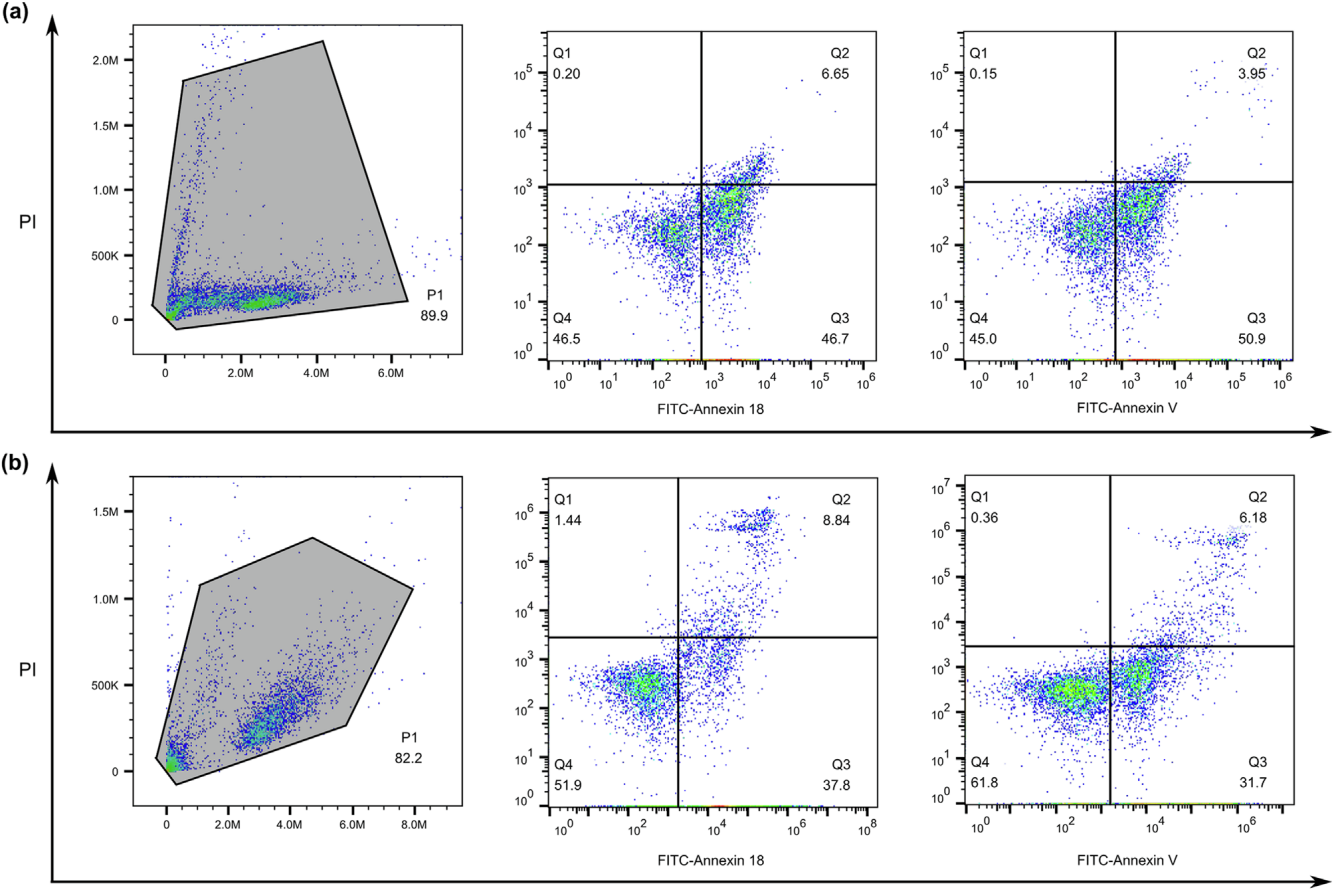
Flow cytometric analysis of SmANX 18 or annexin A5 staining. (a) Representative dot plots of double-stained Jurkat apoptotic cells were assayed by FITC-annexin 18/PI or FITC-annexin A5/PI binding. (b) The double-parameter apoptotic cell scatter diagram of RAW264.7 cells was evaluated by FITC-annexin 18/PI or FITC-annexin A5/PI binding. Ten thousand events were collected for each histogram. The bottom left region of each quadrant (Q4) shows normal living cells, which exclude PI and are negative for FITC-annexin 18 (or A5) binding. The bottom right region (Q3) represents the early apoptotic cells, FITC-annexin 18 (or A5)-positive and PI-negative, demonstrating cytoplasmic membrane integrity. The top right region (Q2) contains necrotic or late apoptotic cells, both positive for FITC-annexin 18 (or A5) binding and PI. The top left region (Q1) may be cell fragments without a cell membrane or dead cells caused by other reasons.

SmANX18 was labelled with fluorescein isothiocyanate (FITC), and then apoptotic cells with FITC-annexin 18 were measured by flow cytometry. During the assay, early apoptotic cell staining was evaluated using FITC-annexin 18 (green fluorescence), while costaining with propidium iodide (PI) was used to discriminate late apoptotic and necrotic cells (red fluorescence). As shown in the FITC-annexin/PI display system, cells in Q2 were FITC+/PI+ cells (late withering or necrotic cell group), Q3 represented FITC+/PI- cells (early withering cell group), and the Q4 quadrant represented FITC-/PI- cells (normal cell group). In the apoptotic state, the double-stained human Jurkat T lymphocytic cell line showed typical flow cytometric dot plots (Fig. 7a). After culture with serum-free medium for 12 h, FITC–annexin 18/PI binding analysis showed that the percentages of the early apoptotic cell population (FITC+PI−) and the late apoptotic cell population (FITC+PI+) were 46.7% and 6.65%, respectively. Similarly, FITC–annexin V/PI binding analysis revealed that early apoptotic cells and late apoptotic cells accounted for 50.9% and 3.95%, respectively. Another apoptotic model of 10% ethanol-induced apoptosis was investigated using the RAW264.7 cell line. The early apoptosis rates of cells incubated with FITC-annexin 18 and FITC-annexin V were 37.8% and 31.7%, respectively as determined by flow cytometry analysis, while the late apoptosis rates using PI staining were 8.84% and 6.18%, respectively (Fig. 7b). The assay showed that the early apoptosis rates of two cell lines stained with FITC-annexin 18 were similar to those stained with FITC-annexin V, indicating that SmANX 18 has the ability to recognize early apoptotic cells. The early apoptosis rate of RAW264.7 cells using FITC-annexin 18 was higher than that of cells using FITC-annexin V, suggesting that SmANX 18 has a greater ability to combine with apoptotic cells of certain mammalian tumor cell lines.

### ANXs in Platyhelminthes of medical interest

**Figure S2:**
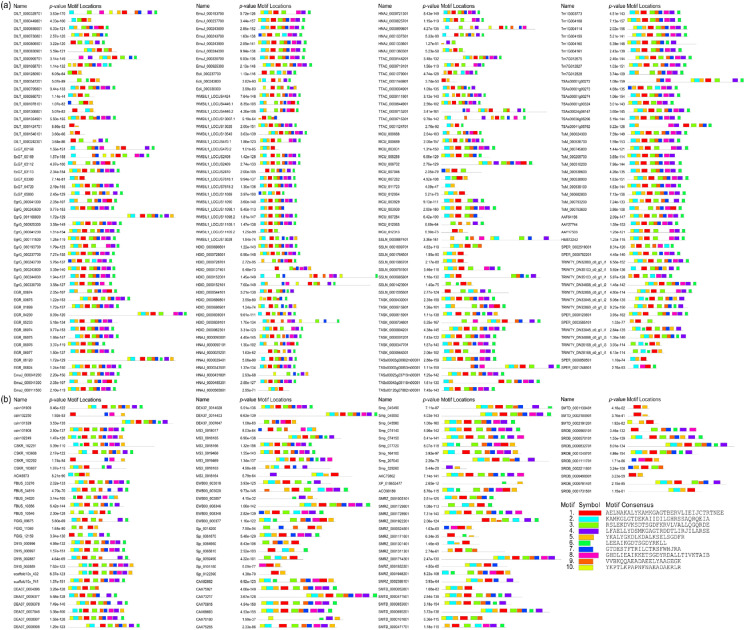
Conserved ANX sequence motifs in medically relevant Platyhelminthes.

A total of 298 ANX sequences in 15 cestodes and 11 trematodes were retrieved from public databases (Supplementary Table S2). The number of ANX genes varied among different species. In the Diphyllobothriidea of Cestoda, 48 members were screened: 18 in *D. latus*, 18 in *S. erinaceieuropaei*, and 8 in *S. solidus*. Most ANX members were found in the family Taeniidae: 44 in the *Echinococcus* tapeworms, 43 in the *Taenia* genus, and 10 in *Hydatigera taeniaeformis.* In the Hymenolepididae, 41 ANXs were screened, while only 14 members were found in Mesocestoididae. Within the medically relevant trematodes, 102 ANX sequences were retrieved, most of which focused on the family Schistosomatidae (69 members). There were 10, 14, and 9 ANX genes in Opisthorchiidae, Fasciolidae and Troglotrematidae, respectively. The MEME program determined 10 highly conserved specific putative motifs that contain 17–33 residues from all sequences. The motif permutation 10 + 2 + 5 + 1 + 3 + 9 + 7 + 8 + 4 + 6 was the most common and widely distributed in cestode and trematode species*.* In addition, a specific single motif containing the “endonexin fold”, such as motifs 1, 2, and 4, was identified in several motif permutations.For the cestodes, among a total of 54 motif permutations, the most frequently appearing motif combination was the motif permutation of 2 + 5 + 1 + 3 + 9 + 7 + 8 + 4 + 6, followed by motifs 2 + 3 + 8 + 10 + 5 + 1 + 9 + 7 + 4 + 6 and 10 + 2 + 5 + 1. For trematodes, the combination of 10 + 2 + 5 + 1 + 3 + 9 + 7 + 6 + 4 appeared most frequently, followed by 10 + 2 + 6 + 3 + 9 + 7 + 8 + 1 + 5 (Supplementary Fig. S2).

### Phylogenetic trees

**Figure 8:**
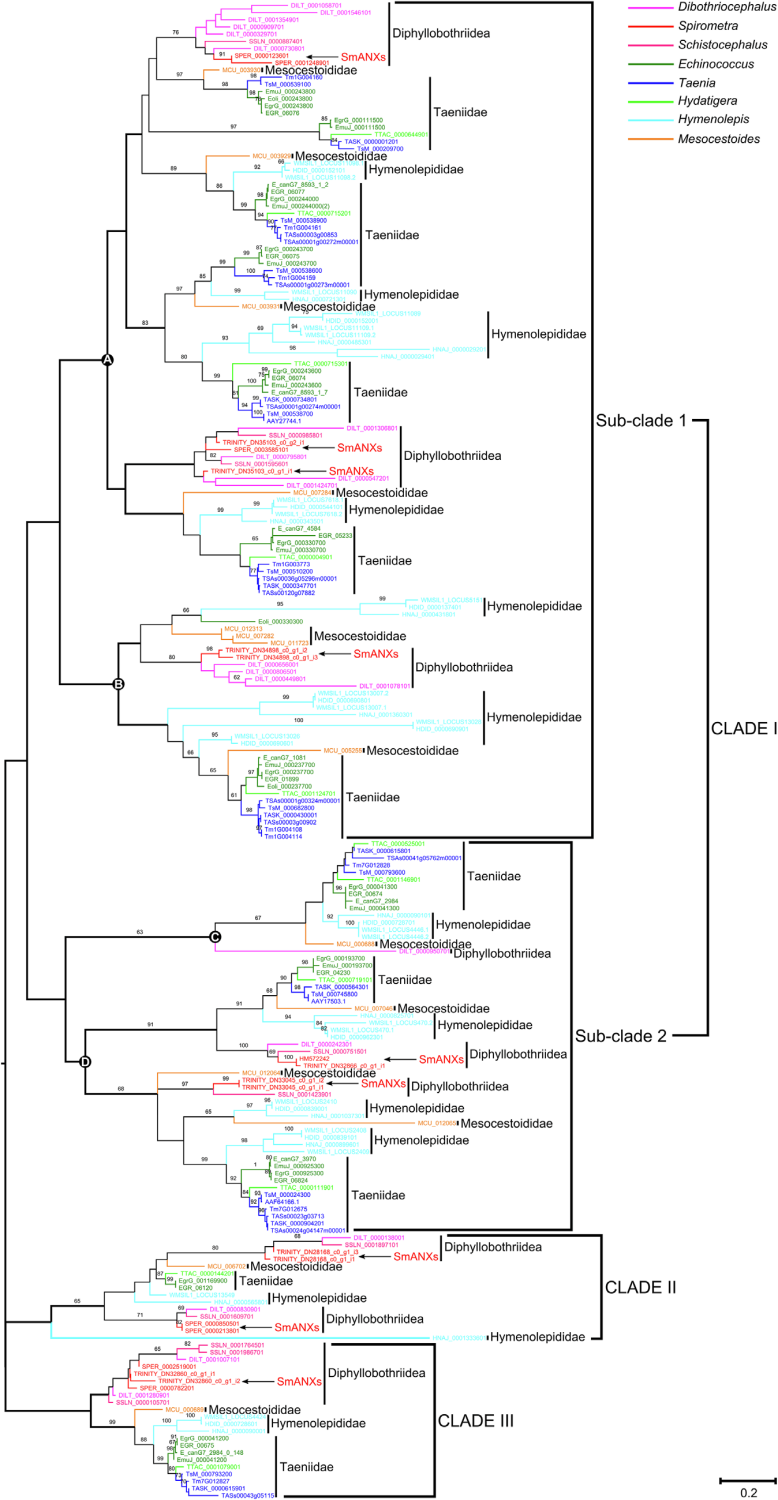
Phylogenetic analysis of annexin sequences in selected medical cestodes based on the maximum likelihood method. The annexin sequences from *S. mansoni* are indicated by arrows. The numbers on the branches represent bootstrap values, and only values with bootstrap values >60 are displayed.

**Figure S3:**
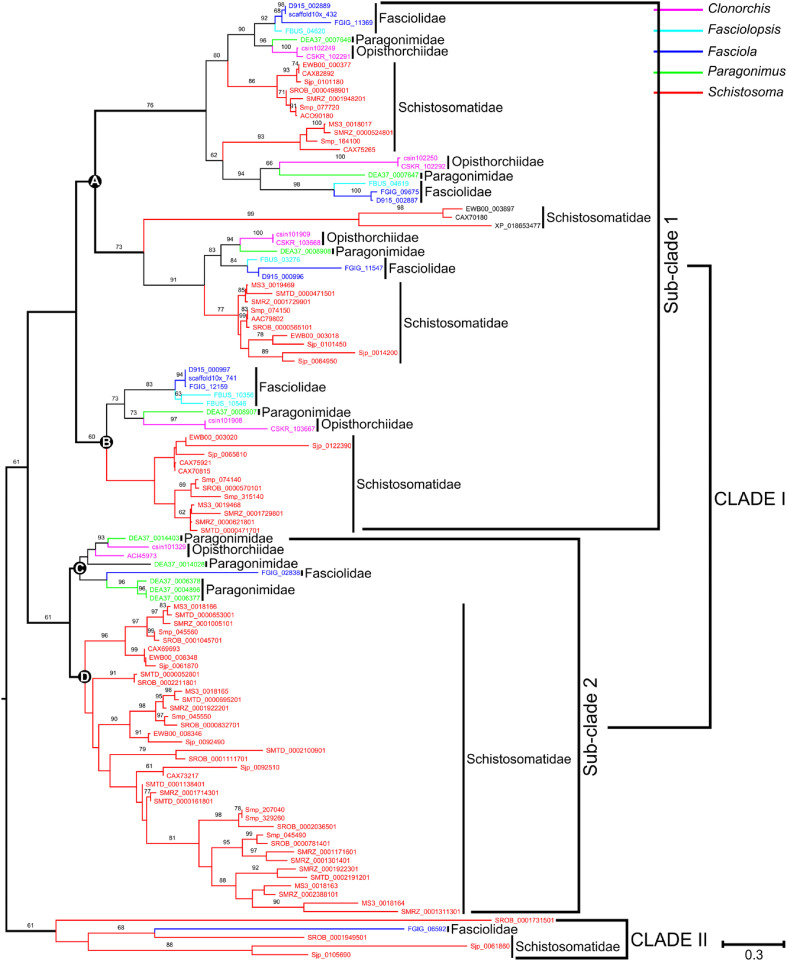
Phylogenetic trees showing the evolutionary relationship of annexin proteins from trematodes of medical interest by the maximum likelihood method. The annexin sequences from *S. mansoni* are indicated by arrows.

**Figure S4:**
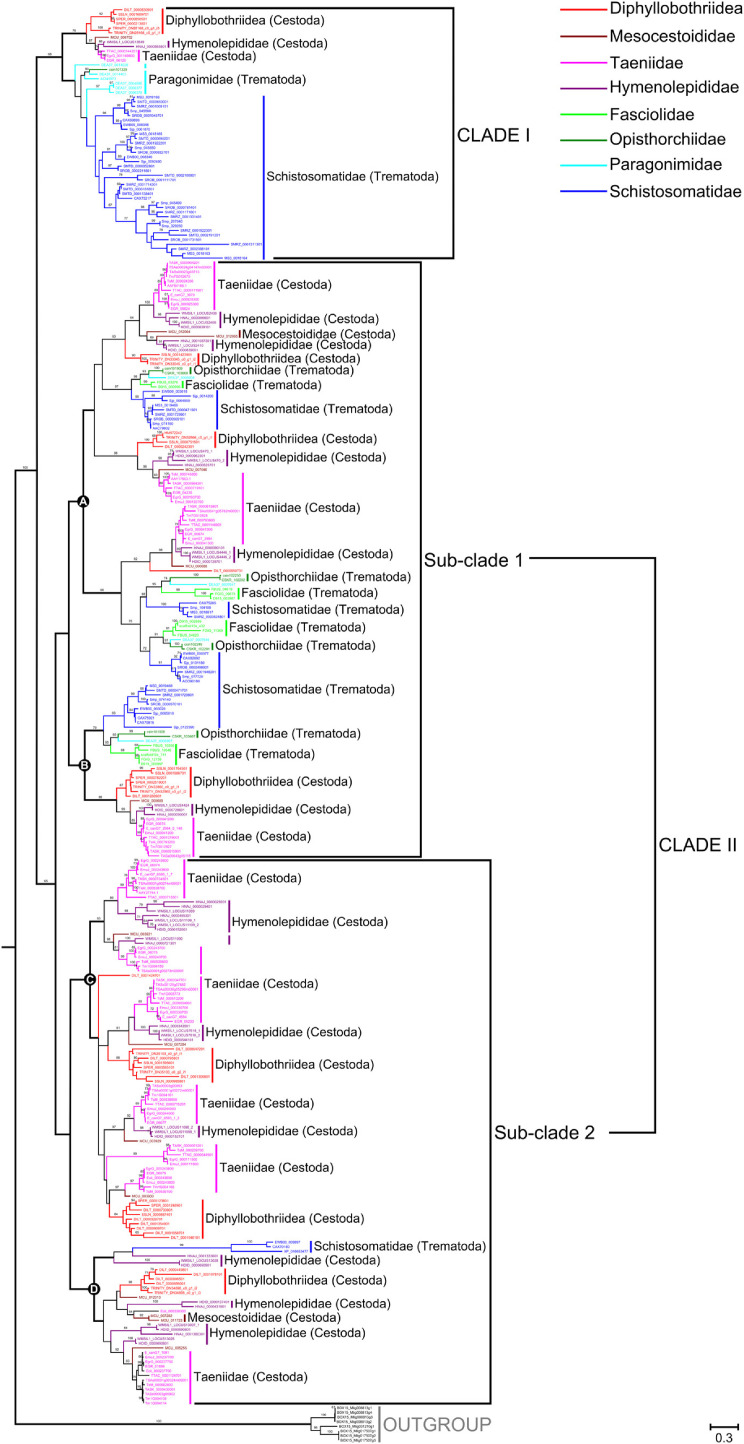
Phylogenetic relationships of the annexin protein family among medically relevant helminths (cestodes + trematodes). The phylogenetic tree was generated with the maximum likelihood method. The numbers on the branches represent bootstrap values, and only values with bootstrap values >60 are displayed.

Among cestodes, although three main clades (Clades I, II, and III) were revealed, no tapeworm groups clustered according to their taxonomic systems, and species in all four medical tapeworm families, Diphyllobothriidea, Taeniidae, Hymenolepididae, and Mesocestoididae, were dispersed in each generated clade (Fig. 8). According to the phylogenetic analysis, most cestodes clustered with Clade I, which can be divided into two subclades: Subclades 1 and 2. Each subclade was composed of two separated groups (Groups A and B in subclade 1 and Groups C and D in subclade 2). Interestingly, species in all four families were still dispersed in each group, while they tended to group together on the basis of their taxonomic units; that is, tapeworms in the same genus or family tended to group together. Similar phylogenetic patterns were also found in Clades II and III. For *S. mansoni*, its members were scattered in all three clades, and even each group within Clade I. For the trematodes, members in four families (Opisthorchiidae, Fasciolidae, Troglotrematidae, and Schistosomatidae) were included in the phylogenetic analysis. The ML analysis generated two main clades: Clades I and II, with the overwhelming majority of samples clustered in Clade I (Supplementary Fig. S3). Trematodes within Clade I can be classified into two subclades (Subclades 1 and 2), with each subclade consisting of two independent groups. Consistent with phylogenetic patterns in cestodes, flukes in Subclade 1 were dispersed in each of the individual groups. However, within Subclade 2, species belonging to Opisthorchiidae, Fasciolidae, and Troglotrematidae made up a single group (Group C), while several *Schistosoma* trematodes formed the other group (Group D). When the datasets of both cestodes and trematodes were combined, the tree topology revealed two main clades (Clades I and II) (Supplementary Fig. S4). Generally, all obtained Platyhelminthes were dispersed in each generated cluster in the phylogenetic tree, consistent with patterns shown in the separate analysis of cestodes and trematodes. Within Clade I, cestodes in Diphyllobothriidea, Hymenolepididae, and Taeniidae made up a single group, while trematodes in Troglotrematidae and Schistosomatidae formed the other group. Clade II was further divided into Subclades 1 and 2. Within Subclade 1, cestodes and trematodes crossed the distribution in Group A. In Group B, trematodes belonging to Schistosomatidae, Opisthorchiidae, and Fasciolidae clustered together, and cestodes in Diphyllobothriidea, Hymenolepididae, and Taeniidae formed the other cluster. Within Subclade 2, except for a few *Schistosoma* trematodes, all members were tapeworms.

## Discussion

It is well known that the wide distribution of annexin members in parasites indicates their importance in various roles in cellular physiology, which may be important for parasitism [37]. Although several tapeworm annexins have been studied in detail [29, 42], their family structures, molecular characterizations, and evolutionary patterns have received little attention in the neglected tapeworm *S. mansoni*.

In this study, 18 new annexin members in *S. mansoni* were identified for the first time. The subcellular localization prediction combined with NCBI database searches revealed that 17 members belong to intracellular ANXs (11 cANXs and 6 mANXs), and only one secretory annexin was found. Most members of the annexin family were found to be distributed in the cytoplasm [51]. In rare cases, a few annexins without the signal peptide can be secreted into the extracellular space to exert their physiological effects through non–classical secretion pathways (e.g., annexin A2 and A5 of humans and annexin B3 of *E. granulosus*) [46, 47]. The clustering analysis demonstrated that 18 SmANXs were divided into two main classes, consistent with the patterns of conserved motif organization. The 3D structure analysis showed that the central domain of SmANX consisted of four repetitive sequences containing calcium-binding sites, which indicated that annexins bind phospholipids in a Ca^2+^-dependent manner to exert their various functions [5]. A total of 18 SmANXs were detected at all developmental stages (plerocercoid, adult, and egg) and displayed ubiquitous but highly variable expression patterns in all tissues/organs studied, indicating functional divergence of annexin members in *S. mansoni*.

To explore the molecular characteristics of native SmANX, one representative sequence (SmANX18) was selected for cloning in an *E. coli* expression system. Purified rSmANX induced strong specific antibodies against rSmANX in BALB/c mice and therefore can be used as an immunogen to produce antibodies. Previous studies have suggested that parasites communicate with the host through the tegument, and molecules distributed in the tegument are important in the host-parasite interaction [46, 49]. IFA analysis showed that SmANXs were located in the tegument and parenchyma in both the plerocercoid and adult stages, indicating that these molecules probably play roles in host-parasite interactions. In addition, the annexin distributed in the tegument of the worm had strong immunogenicity and unique structural features [38, 41], which indicated that SmANX could be expected to play an antiparasitic role as a vaccine and drug target in the future. qPCR results showed that the selected SmANX was transcribed at various stages of *S. mansoni* and highly expressed in the immature proglottid of adults, indicating wide participation of annexins in the development and growth of the parasite. Moreover, the study of Hofmann et al. [19] indicated that annexins can protect parasite tissues and help them escape attack from the host immune system during parasitism. Interestingly, fluorescence staining was observed in the subcutaneous region of plerocercoids, implying potential roles of SmANX during the process of plerocercoid invasion into the host.

Annexins can bind phospholipids with high-affinity in a Ca^2+^-dependent manner, playing a unique and important role in the regulation of many biological processes [43]. In this study, rSmANX18 could bind to phospholipid membranes in the presence of calcium ions, indicating that rSmANX has calcium-dependent membrane binding properties, and this characteristic of membrane-associated proteins has also been confirmed in *Schistosoma mansoni* and *Cysticercus cellulosae* [53]. Recently, the finding that hemorrhage and thrombosis are key factors leading to clinical deterioration and death prompted global attention to relevant anticoagulants [44]. As an anticoagulant protein, annexin has attracted more discussion about its anticoagulant effect and related mechanisms. Previous studies of the anticoagulant activities of parasite annexins have mainly focused on limited species, such as *T. solium* and *S. bovis* [54]. Here, the assay using both the slide method and aPTT method confirmed that SmANX possesses anticoagulant activity and could play a role in the exogenous coagulation pathway. For annexin in *S. mansoni*, we inferred that the anticoagulant activity of annexin may be of great significance for the migration and parasitism of plerocercoids in the host. Apoptosis is crucial in the whole growth process of organisms, contributing to the renewal of tissues and the elimination of inflammatory cells [39]. As well documented, phosphate serine (PS) is usually transferred to the outer surface of the cell membrane during apoptosis, which can be quantified by the binding of FITC-Annexin [26]. At present, the annexin A5-binding assay is routinely used for laboratory and clinical studies [11]. However, the cost of purchasing this reagent limits the application of this assay. In this study, we first prepared SmANX as a detection reagent to identify apoptotic cells from the RAW264.7 and Jurkat cell lines. The results showed that FITC-SmANX was as sensitive as FITC-annexin A5 in detecting PS exposure in different apoptotic cells and even better for recognizing a specific mammalian tumor cell line, suggesting that SmANX is promising as a practical tool for detecting early apoptosis in certain cell lines in the future.

The number of ANX family members varied among different species of medically revelant Platyhelminthes, indicating the genetic diversity of annexin genes. Nevertheless, a conserved motif permutation was identified among most cestode and trematode species, suggesting the conservativeness of ANXs in Platyhelminthes. During the phylogenetic analysis, the annexin members of Platyhelminthes were grouped into two main clades; however, no groups clustered according to their taxonomic systems. Cestode and trematode species were dispersed in each generated clade, indicating that there are probably two main genotypes of annexin in Platyhelminthes, and the two genotypes are indispensable for both cestodes and trematodes. More interestingly, annexins in each specific species group (such as the same family) tended to cluster together, suggesting the similarity of ANX in closely related species. In *S. mansoni*, ANX family members were found to be scattered in different subclades, confirming the genetic polymorphism of SmANXs.

## Conclusions

In this study, we first identified 18 new ANX members in *S. mansoni* and investigated their expression patterns at different developmental stages. Next, a family member was selected to explore molecular characteristics. rSmANX was successfully cloned and expressed. The protein was immunolocalized in the tegument and parenchyma of the plerocercoid and in the tegument, parenchyma, uterus, and egg shell of adult worms. rSmANX can bind with phospholipids with high-affinity in a Ca^2+-^dependent manner, and the recombinant protein showed high anticoagulant activity and could combine with FITC to recognize apoptotic cells. Finally, a phylogenetic analysis was performed to investigate the evolutionary pattern of ANXs across medically relevant Platyhelminthes. Gene polymorphism and conservative core motif permutation were found in both cestodes and trematodes. SmANXs revealed high genetic diversity. The findings of this study will lay a foundation for further studies on the biological function of ANXs in *S. mansoni* as well as other taxa in which ANXs occur*.*

## Abbreviations

ANX: annexin
cox1: cytochrome c oxidase subunit 1
SPF: specific pathogen-free
IP: immature proglottid
MP: mature proglottid
GP: gravid proglottid
ORF: open reading frame
PCR: polymerase chain reaction
aPTT: activated partial thromboplastin time
CT: coagulation time
FITC: fluorescein isothiocyanate
ML: maximum likelihood
BI: Bayesian inference

## Data Availability

The data supporting the conclusions of this article are included within the article.

## Supplementary materials

The supplementary material for this article can be found at https://www.parasite-journal.org/10.1051/parasite/2024034/olm.

*Table S1*: Primers used in qRT‒PCR analysis. *Table S2*: Summary of ANXs in other helminths. *Table S3*: Putative motifs of SmANXs.
