# Supplementary material for: Annexin gene family in Spirometra mansoni (Cestoda: Diphyllobothriidae) and its phylogenetic pattern among Platyhelminthes of medical interest
Source: Parasite. 2024 Jun 21;31:32. doi: 10.1051/parasite/2024034 (PMC11195529; doi:10.1051/parasite/2024034)
Supplement: Supplementary file 1 — Table S1: Primers used in qRT‒PCR analysis. Table S2: Summary of ANXs in other helminths. Table S3: Putative motifs of SmANXs. [file parasite-31-32-s1.zip › parasite230101-1-olm/Table S1.pdf]

Table S1. Primers used in qRT–PCR analysis

Gene	Primer name	Sequence (5'-3')	Product size (bp)
SPER_0000850501	SPER_0000850501-S<br>SPER_0000850501-A	GTGACCTACGCCTTGGAT<br>CAAGTGACTGCCCGTTTAT	297
SPER_0002519001	SPER_0002519001-S<br>SPER_0002519001-A	AGTATGCCAAAGTGCTCC<br>CACCCTTGTTGAGCATAGTT	171
SPER_0000123601	SPER_0000123601-S<br>SPER_0000123601-A	TACAAGGGTGGCGAAGCG<br>GCCGACCGCCAATACACT	135
SPER_0000782201	SPER_0000782201-S<br>SPER_0000782201-A	AGTATGCCAAAGTGCTCC<br>TCATCTGTTCCAAGACCCT	184
SPER_0003585101	SPER_0003585101-S<br>SPER_0003585101-A	GCAGTGCCGTTGTATGGT<br>GTGCGATTAGGACTCGTTTG	105
SPER_0001248901	SPER_0001248901-S<br>SPER_0001248901-A	TGCCCTTTCCAGTTCCAT<br>CCGACCGCCTTTCCGTAT	156
DN26168_c0g1i3	DN26168_c0g1i3-S<br>DN26168_c0g1i3-A	TCAAGTGAGCGTTGGAGC<br>CGACAGGAACAAGGCAAG	119
DN26168_c0g1i1	DN26168_c0g1i1-S<br>DN26168_c0g1i1-A	TCAAGTGAGCGTTGGAGC<br>TTGTTTGGCTCCGTTGCT	80
DN34898_c0g1i2	DN34898_c0g1i2-S<br>DN34898_c0g1i2-A	AGCCTTATGAGGTCCTTGTC<br>CGTTGGTCGCAATCGTT	109
DN34898_c0g1i3	DN34898_c0g1i3-S<br>DN34898_c0g1i3-A	ACGAAACTGCGTTGGGT<br>TCCTGGTTGCTCTGCTCC	157
DN32860_c0g1i2	DN32860_c0g1i2-S<br>DN32860_c0g1i2-A	GGTGAGGCGGTAGGTTAG<br>GACGGCACAAATTAGTCG	175
DN33045_c0g1i2	DN33045_c0g1i2-S<br>DN33045_c0g1i2-A	GTCAGAGTCAACATCGCCTTC<br>TCGCCACCTGCTCAAACG	113
DN32866_c0g1i1	DN32866_c0g1i1-S<br>DN32866_c0g1i1-A	GATACATCGGCGACTCAG<br>CGTCAACAGATAAAGGAGGT	124
DN35103_c0g1i1	DN35103_c0g1i1-S<br>DN35103_c0g1i1-A	TTGTTTGGCTCCGTTGCT<br>CGTTTGGTCTTGGCGTAC	172
DN33045_c0g1i1	DN33045_c0g1i1-S<br>DN33045_c0g1i1-A	GTCAGAGTCAACATCGCCTTC<br>TCGCCACCTGCTCAAACG	113
DN32860_c0g1i1	DN32860_c0g1i1-S<br>DN32860_c0g1i1-A	GAGTCAGGTAGGCGTTCTT<br>GACGGCACAAATTAGTCG	149
DN35103_c0g2i1	DN35103_c0g2i1-S<br>DN35103_c0g2i1-A	CTCGTCAGTTCCCACCTT<br>GAGTCCTAATCGCACTTCTT	116
HM572242	HM572242-S<br>HM572242-A	CCAACCATTACCCCATCCCC<br>TTGACGCTCCTCACTGGTTC	135
GAPDH	GAPDH-S<br>GAPDH-A	AGCAACCTCGTTGATGTCGT<br>TGAATTGACCGTGGGTGGAG	97
