# Supplementary material for: Annexin gene family in Spirometra mansoni (Cestoda: Diphyllobothriidae) and its phylogenetic pattern among Platyhelminthes of medical interest
Source: Parasite. 2024 Jun 21;31:32. doi: 10.1051/parasite/2024034 (PMC11195529; doi:10.1051/parasite/2024034)
Supplement: Supplementary file 1 — Table S1: Primers used in qRT‒PCR analysis. Table S2: Summary of ANXs in other helminths. Table S3: Putative motifs of SmANXs. [file parasite-31-32-s1.zip › parasite230101-1-olm/Table S2.pdf]

Table S2. Summary of ANXs in other helminthes

Class	Family	Genus	SPECIES	SS	SEQUENCE ID
Cestoda	Diphyllobothriidea	*Dibothriocephalus*	*D. latus*	20	DILT_0001058701,<br>DILT_0000730801,<br>DILT_0000909701,<br>DILT_0000329701,<br>DILT_0001280901,<br>DILT_0000806501,<br>DILT_0000449801,<br>DILT_0000656001,<br>DILT_0000830901,<br>DILT_0001306801,<br>DILT_0001546101,<br>DILT_0000950701,<br>DILT_0000795801,<br>DILT_0000242301,<br>DILT_0000547201,<br>DILT_0001354901,<br>DILT_0001007101,<br>DILT_0000138001,<br>DILT_0001424701,<br>DILT_0001078101
		Spirometra	*S. erinaceieuropaei*	18	SPER_0000123601,<br>SPER_0002519001,<br>SPER_0000782201,<br>SPER_0003585101,<br>SPER_0000850501,<br>SPER_0001248901,<br>DN28168_c0_g1_i3,<br>DN34898_c0_g1_i2,<br>DN28168_c0_g1_i1,<br>DN33045_c0_g1_i2,<br>DN32860_c0_g1_i2,<br>DN34898_c0_g1_i3,<br>DN32866_c0_g1_i1,<br>DN35103_c0_g2_i1,<br>DN32860_c0_g1_i1,<br>DN35103_c0_g1_i1,<br>DN33045_c0_g1_i1,<br>HM572242
		Schistocephalus	*S. solidus*	10	SSLN_0001986701,<br>SSLN_0001764501,<br>SSLN_0000887401,<br>SSLN_0001609701,<br>SSLN_0001595601,<br>SSLN_0000751501,<br>SSLN_0000985801,<br>SSLN_0001423901,<br>SSLN_0001897101,

					SSLN_0000105701
	Taeniidae	*Echinococcus*	*E. canadensis*	7	EcG7_00168,<br>EcG7_05893,<br>EcG7_03380,<br>EcG7_03112,<br>EcG7_00169,<br>EcG7_04720,<br>EcG7_03113
			E. granulosus	24	EgrG_000243800,<br>EgrG_000193700,<br>EgrG_000243700,<br>EgrG_000111500,<br>EgrG_000925300,<br>EgrG_000330700,<br>EgrG_000041300,<br>EgrG_000243600,<br>EgrG_000244000,<br>EgrG_000237700,<br>EgrG_000041200,<br>EgrG_001169900<br>EGR_04230,<br>EGR_06120,<br>EGR_06824,<br>EGR_06076,<br>EGR_00674,<br>EGR_06075,<br>EGR_01899,<br>EGR_06074,<br>EGR_05233,<br>EGR_06077,<br>EGR_00675
			E. multilocularis	11	EmuJ_000243800,<br>EmuJ_000193700,<br>EmuJ_000243700,<br>EmuJ_000111500,<br>EmuJ_000925300,<br>EmuJ_000244000,<br>EmuJ_000330700,<br>EmuJ_000041300,<br>EmuJ_000237700,<br>EmuJ_000243600,<br>EmuJ_000041200
			E. oligarthrus	3	Eoli_000237700,<br>Eoli_000330300,<br>Eoli_000243800
		Taenia	*T. asiatica*	13	TASs00003g00902,<br>TASs00043g05115,<br>TASs00120g07882,<br>TASs00023g03713,

					TASs00003g00853, TASK_0000904201, TASK_0000615801, TASK_0000430001, TASK_0000615901, TASK_0000001201, TASK_0000564301, TASK_0000347701, TASK_0000734801
			T. multiceps	9	Tm7G012828, Tm1G004160, Tm1G004159, Tm7G012675, Tm1G003773, Tm1G004161, Tm1G004114, Tm1G004108, Tm7G012827
			T. saginata	7	TSAs00001g00273, TSAs00041g05762, TSAs00036g05296, TSAs00024g04147, TSAs00001g00324, TSAs00001g00272, TSAs00001g00274
			T. solium	14	TsM_000024300, TsM_000745800, TsM_000538700, TsM_000538600, TsM_000539100, TsM_000209700, TsM_000510200, TsM_000538900, TsM_000793600, TsM_000793200, TsM_000682800, AAF64166.1, AAY27744.1, AAY17503.1
		Hydatigera	*H. taeniaeformis*	11	TTAC_0000719101, TTAC_0000111901, TTAC_0001146901, TTAC_0001079001, TTAC_0000144201, TTAC_0001124701, TTAC_0000715201, TTAC_0000644901, TTAC_0000715301, TTAC_0000004901,

					TTAC_0000525001
	Hymenolepididae	*Hymenolepis*	*H. diminuta*	28	WMSIL1_LOCUS13007, WMSIL1_LOCUS4446, WMSIL1_LOCUS4424, WMSIL1_LOCUS13026, WMSIL1_LOCUS13549, WMSIL1_LOCUS2408, WMSIL1_LOCUS11089, WMSIL1_LOCUS7618, WMSIL1_LOCUS13028, WMSIL1_LOCUS470, WMSIL1_LOCUS11090, WMSIL1_LOCUS2409, WMSIL1_LOCUS2410, WMSIL1_LOCUS11098, WMSIL1_LOCUS11109, WMSIL1_LOCUS5151, HDID_0000728701, HDID_0000690601, HDID_0000728601, HDID_0000152101, HDID_0000839101, HDID_0000690801, HDID_0000962301, HDID_0000690901, HDID_0000839001, HDID_0000544101, HDID_0000152001, HDID_0000137401
			H. nana	14	HNAJ_0000090001, HNAJ_0000090101, HNAJ_0001360301, HNAJ_0001333601, HNAJ_0000825701, HNAJ_0000029201, HNAJ_0000029401, HNAJ_0000899601, HNAJ_0001037301, HNAJ_0000485301, HNAJ_0000721301, HNAJ_0000431801, HNAJ_0000343501, HNAJ_0000565801
	Mesocestoididae	*Mesocestoides*	*M. corti*	14	MCU_012064, MCU_000688, MCU_011723, MCU_007282, MCU_003931, MCU_005255,

					MCU_000689, MCU_007046, MCU_006702, MCU_003929, MCU_003930, MCU_007284, MCU_012313, MCU_012065
Trematoda	Opisthorchiidae	*Clonorchis*	*C. sinensis*	10	csin102249, csin101908, csin101909, csin101329, csin102250, ACI45973, CSKR_102292, CSKR_103668, CSKR_102291, CSKR_103667
	Fasciolidae	*Fasciolopsis*	*F. buski*	5	FBUS_04620, FBUS_04619, FBUS_10356, FBUS_10546, FBUS_03276
		Fasciola	*F. gigantica*	6	FGIG_09675, FGIG_12159, FGIG_02838, FGIG_11547, FGIG_11369, FGIG_06592
			F. hepatica	6	D915_002887, D915_000997, D915_000996, D915_002889, maker-scaffold10x_432, maker-scaffold10x_741
	Troglotrematidae	*Paragonimus*	*P. westermani*	9	DEA37_0007646, DEA37_0008907, DEA37_0008908, DEA37_0014028, DEA37_0004896, DEA37_0006377, DEA37_0006378, DEA37_0014403, DEA37_0007647
	Schistosomatidae	*Schistosoma*	*S. haematobium*	7	MS3_0019468, MS3_0018017, MS3_0018165, MS3_0019469, MS3_0018166, MS3_0018164, MS3_0018163
			S. japonicum	24	Sjp_0014200,

					Sjp_0061860, Sjp_0061870, Sjp_0064950, Sjp_0065810, Sjp_0092490, Sjp_0092510, Sjp_0101180, Sjp_0101450, Sjp_0105690, Sjp_0122390,CAX75265, CAX70180, CAX70815, CAX69693, CAX82892, CAX75921, CAX73217, EWB00_000377, EWB00_003897, EWB00_003020, EWB00_003018, EWB00_008348, EWB00_008346
			S. mansoni	13	Smp_074150, Smp_074140, Smp_164100, Smp_045490, Smp_077720, Smp_045560, Smp_045550, Smp_207040, Smp_329260, Smp_315140, XP_018653477, AAC79802, ACO90180
			S. margrebowiei	13	SMRZ_0000621801, SMRZ_0001729901, SMRZ_0001729801, SMRZ_0001005101, SMRZ_0001922201, SMRZ_0000524801, SMRZ_0001301401, SMRZ_0002388101, SMRZ_0001948201, SMRZ_0001311301, SMRZ_0001922301, SMRZ_0001714301, SMRZ_0001171601
			S. mattheei	9	SMTD_0000471501, SMTD_0000052801, SMTD_0000653001, SMTD_0000695201, SMTD_0002100901,

					SMTD_0000471701, SMTD_0000161801, SMTD_0001138401, SMTD_0002191201
			S. rodhaini	11	SROB_0001111701, SROB_0000565101, SROB_0000570101, SROB_0001045701, SROB_0002211801, SROB_0000832701, SROB_0001949501, SROB_0000781401, SROB_0000498901, SROB_0002036501, SROB_0001731501
Turbellaria	Macrostomidae	*Macrostomum*	*M. lignano*	8	BOX15_Mlig008813g1, BOX15_Mlig008813g2, BOX15_Mlig008813g3, BOX15_Mlig017507g3, BOX15_Mlig031210g1, BOX15_Mlig017507g1, BOX15_Mlig017507g2, BOX15_Mlig008813g4
