# Supplementary material for: Annexin gene family in Spirometra mansoni (Cestoda: Diphyllobothriidae) and its phylogenetic pattern among Platyhelminthes of medical interest
Source: Parasite. 2024 Jun 21;31:32. doi: 10.1051/parasite/2024034 (PMC11195529; doi:10.1051/parasite/2024034)
Supplement: Supplementary file 1 — Table S1: Primers used in qRT‒PCR analysis. Table S2: Summary of ANXs in other helminths. Table S3: Putative motifs of SmANXs. [file parasite-31-32-s1.zip › parasite230101-1-olm/Table S3.pdf]

Table S3. Putative motifs of SmANXs

Motif	Consensus sequence	Length	No. of annexin proteins	e-value
1	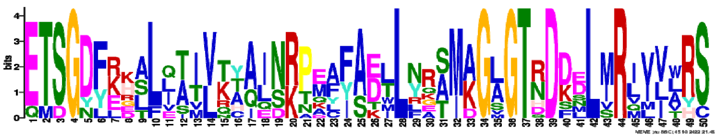	50	16	6.2e-310
2	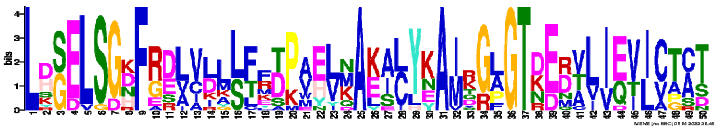	50	15	3.9e-278
3	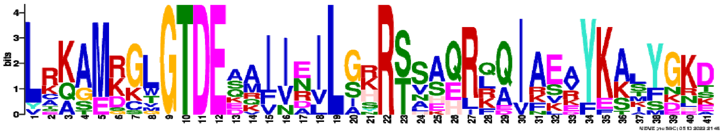	41	16	1.7e-219
4	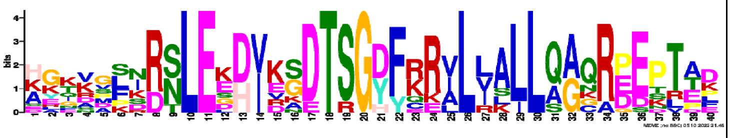	40	16	8.4e-211
5	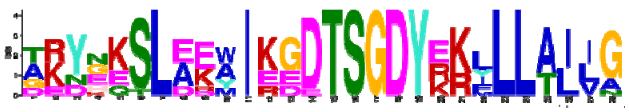	28	12	5.5e-118
6	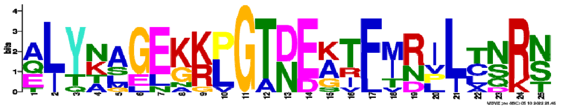	25	11	5.8e-062
7	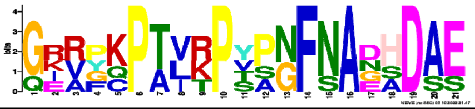	21	9	1.6e-058
8	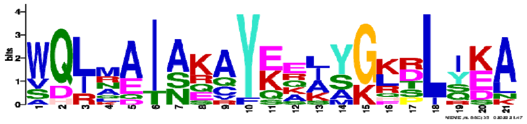	21	12	1.3e-037
9	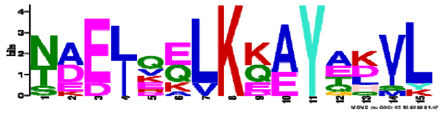	15	12	3.8e-028
10	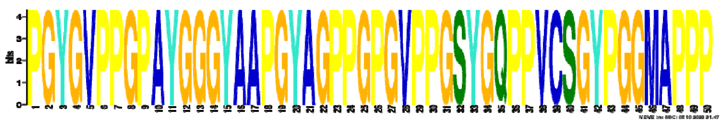	50	2	5.2e-025
